# Impaired GABA Neural Circuits Are Critical for Fragile X Syndrome

**DOI:** 10.1155/2018/8423420

**Published:** 2018-10-03

**Authors:** Fei Gao, Lijun Qi, Zhongzhen Yang, Tao Yang, Yan Zhang, Hui Xu, Huan Zhao

**Affiliations:** ^1^Department of Anesthesiology, Heze Municipal Hospital, Heze, 274031 Shandong, China; ^2^Department of Pain Treatment, Tangdu Hospital, Fourth Military Medical University, Xi'an 710038, China; ^3^Department of Neurobiology and Collaborative Innovation Center for Brain Science, School of Basic Medicine, Fourth Military Medical University, Xi'an 710032, China

## Abstract

Fragile X syndrome (FXS) is an inheritable neuropsychological disease caused by silence of the *fmr1* gene and the deficiency of Fragile X mental retardation protein (FMRP). Patients present neuronal alterations that lead to severe intellectual disability and altered sleep rhythms. However, the neural circuit mechanisms underlying FXS remain unclear. Previous studies have suggested that metabolic glutamate and gamma-aminobutyric acid (GABA) receptors/circuits are two counter-balanced factors involved in FXS pathophysiology. More and more studies demonstrated that attenuated GABAergic circuits in the absence of FMRP are critical for abnormal progression of FXS. Here, we reviewed the changes of GABA neural circuits that were attributed to intellectual-deficient FXS, from several aspects including deregulated GABA metabolism, decreased expressions of GABA receptor subunits, and impaired GABAergic neural circuits. Furthermore, the activities of GABA neural circuits are modulated by circadian rhythm of FMRP metabolism and reviewed the abnormal condition of FXS mice or patients.

## 1. Introduction

Fragile X mental retardation protein (FMRP) is widely expressed in neurons and glia in the brain and acts as an “interactor” regulating ribosome stalling, translational control, and synaptic plasticity in brain circuits [1–3]. FMRP contributes to cognition, emotions, and memory through the referred “interactor” role as well. Fragile X syndrome (FXS) patients are deficient of FMRP due to *fmr1* gene silence caused by a CGG trinucleotide amplification on Xq27.3 in the 5′-UTR on chromosome [4]. According to CGG trinucleotide expansion and clinical symptoms, FM allele mutation-related syndromes could be divided into FXS (>200 repeats) and FXTAS (55–199 repeats) during early diagnosis of FXS. For example, methylation-specific quantitative melt analysis (MS-QMA), respectively, identified methylation mosaicism in an additional 15% and 11% of patients in the Chilean and Australian reports, suggesting the presence of a cryptic FM [5, 6]. Other methods include a variety of polymerase chain reaction (PCR) techniques, such as high polymorphism markers for preimplantation genetic diagnosis (PGD) of FXS [7] and two PCR analyses (PCR screening and PCR premutation) [8]. However, it is difficult to draw a solid criterion due to different inclusive criteria, diagnostic methods, and sample sizes within each study. Although frequencies of clinical characteristics were different between ethnicities, especially in Asian and African people, which provided evidence for genetic counseling [9], FXS is still difficult to be diagnosed on the account of a lack of an obvious phenotype at birth and during prepuberty in clinic.

Previous studies have illustrated that FXS is caused by the alteration at multiple levels from mRNA shuttling to synaptic plasticity and behavioral phenotypes [10]. For example, FMRP regulated proteins in the modulation of synaptic plasticity, which maintain spine shape and dynamics [11–13]. The retardation of FMRP leads to abnormal group I metabotropic glutamate receptor (mGluR) signaling, together with the loss of AMPA and NMDA receptors [2], although recently clinical trials targeting on mGluR1 failed in FXR patients [14]. Specifically, the enzymes for GABA synthesis and degradation, GABA membrane transporters, and a GABA receptor scaffolding protein are downregulated in the absence of FMRP [15]. Besides, FMRP absence GABA_A_ receptor *α*1 and *δ* subunits were downregulated in *fmr1* gene knockout mouse and *Drosophila* [15–17]. All these studies suggest a perplexing, yet not well understood, link between GABAergic signaling, abnormal neuronal circuits, and dysfunctional behaviors in both FXS animal models and patients. Among all alterations of phenomenal function deficits, dendritic abnormalities are the most evident structural changes in FXS. FMRP regulates neuronal branching as well as dendritic spine morphology and density [18, 19]. However, it remains unclear whether plastic changes of inhibitory circuits may cause abnormal spine morphology in FXS or vice versa. In this review, we summarized mechanisms on the effects of inhibitory synapse alteration from circuits to molecular interaction.

## 2. Altered GABA Metabolisms in FXS Animal Models and Patients

There have been great progresses in the altered GABA metabolism underlying FXTAS/FXS pathogenesis. Mitochondria provides energy for the cell and the brain using most of the energy among all organs. There is mounting evidence that mitochondrial dysregulation systemically contributes to the decreased cell function, even during the neonatal period of mice, first reported by Rizzo et al. [20]. It is reported that premutated hippocampal neurites contained significantly fewer mitochondria and reduced mitochondria mobility at early stage of differentiation, despite the presence of appreciable FMRP expression [21]. Together, similar significant deficits of mitochondrial dysfunction, induced by Zn levels, were observed in the Zn-rich regions (the hippocampus and cerebellum of premutation carriers), with some of these effects lasting into adulthood [22, 23]. Particularly, in dysregulated GABAergic circuits, mitochondrial dysfunction plays vital role from the aspects of mitochondrial structure, number, membrane permeability, transport, fusion, and fission [21, 24–27]. It is noteworthy that abnormality of mitochondrial structure and function is regulated aberrant expression of microRNAs (miRNA) [28], while few report functions of miRNAs on GABA metabolism in Fragile X syndrome. More work should be needed to illustrate the perplexing role of deregulated miRNA expression profiles within uncommon GABA neural circuits. In a word, abnormalities of mitochondrial dysfunction induced by FMRP deficits altered GABA metabolism, contributing to the etiology of FXS/FXTAS.

In addition, glutamic acid decarboxylase (GAD) or vesicular GABA transporter protein (VGTA) and vesicular glutamate transporter protein (VGLUT) consist of two components of synaptic balance. Increased expression of VGAT relative to VGLUT expression was shown within the medial nucleus of the trapezoid body (MNTB) in FXS [29]. Their mechanisms are necessary to be further explored. In FXS patients, a reduced release of GABA from the GABAergic terminals to the presynaptic GABA_B_ receptors might induce a decreased inhibition of neurotransmitter spillover, which conversely activated mGluR signaling [30]. One mechanism of modulating GABA release involves the synthesis and mobilization of endocannabinoids. Activation of GroupImGluRs enables mobilization of endocannabinoids in the postsynaptic neuron and negatively modulates GABA release through a mechanism known as depolarization-induced suppression of inhibition (DSI) [31]. Therefore, in consideration of endocannabinoid mobilization in the FXS, it is reported that alterations in eCB signaling could contribute to the cognitive dysfunction associated with FXS [32]. But it only demonstrated DHPG-induced eCB-iLTD, without affecting DSI, at low concentrations. Together, relatively high concentrations of cannabinoids could affect neuropsychiatric disorders via inhibition of monoamine oxidase activity [33]. Therefore, the loss of FMRP may selectively affect specific inhibitory circuits and more evidence is needed in exploring. In the developing and mature brain, it is critical for cortical balance of excitatory and inhibitory neurons to be properly synchronized at behaviorally relevant frequencies. And thus, alteration of mGluR signaling and GABA metabolisms in this specific type of interneuron is likely to have wide-reaching effects in developing and mature cortical networks.

## 3. Decreased Expression of GABA Receptor Subunits in FXS Models

The anomalous functions of mGluR-dependent synaptic plasticity have been observed in the hippocampus of *fmr1*-KO mice. Activity-dependent synthesis of FMRP in maintaining forms of synaptic plasticity may be induced by augmented mGluR-LTD in hippocampal neurons [34, 35], while the initiation of long-term potentiation (LTP) is a qualitatively different functional consequence of mGluR1-stimulated protein synthesis at the synapses of the hippocampus where LTD can be induced. Besides, the mGluR theory proposes that stimulation of mGluR1 induces local mRNA translation, resulting in protein synthesis that subsequently enhances the internalization of AMPA receptors [36]. This model predicts that in the absence of FMRP, the increased translation of a subset of mRNAs disturbs receptor internalization dynamics and then exaggerates internalization of AMPA receptors and weakens the synapse. Interestingly, GABA_B1_ and GIRK_2_ internalization also is reported to cause rapid and persistent weakening of GABA_B_-activated GIRK-mediated (GABA_B_-GIRK) currents in FXS [37]. Clearly, the fate of internalized GABA_A_Rs will therefore play a critical role in controlling cell surface receptor levels and hence the efficacy of synaptic inhibition. This may suggest that GABA receptors take internalization process, but its underlying complicated mechanisms still need to be explored. Furthermore, FMRP absence increased steady state surface levels of GABA_A_Rs, showing a dramatic functional effect of increased surface receptor number. The mechanism underlying postendocytic GABA_A_R sorting remains to be demonstrated, and FMRP's particular role in this process is also an area of active research. The impact of FMRP regulation of GABA_A_Rs was recently shown in the hypothalamus, causing decreased food intake and loss of body weight [38]. An unresolved issue is whether FMRP acts to promote recycling of GABA_A_Rs or prevents their lysosomal degradation.

Furthermore, different subunit combination leads to diverse expression patterns of GABA_A_Rs at specific cell surface. Most surface receptor clusters of *γ*2 receptor subunits are synaptic, while GABA_A_Rs containing *α*5 or *β*3 subunit express higher at extrasynaptic. *δ* subunit is exclusively located outside the synapse at perisynaptic and extrasynaptic locations [39, 40]. For example, it is investigated that tonic GABA_A_ currents in the subiculum were downregulated in the *fmr1* knockout mouse relative to wild-type animals [41]. These results were associated with reductions in tonic GABA_A_ receptor subunits. Furthermore, more specific results based on the different GABAR subunits need to be expanded to better identify each function in FXS.

Results from all above pave the way for many interesting avenues of research. First, more work is needed to illustrate the molecular causes of impaired inhibition in FXS. In the *Drosophila* model, limited research available has demonstrated that a GABA_A_ receptor reduction can lead to behavioral impairments. However, other research from FXS models indicated that the mechanism was likely more complicated and possibly indirect due to not only variable GABA metabolisms but also regional specificity [40, 42, 43]. For example, vision process is modulated by different GABA receptors in spread brain via tonic inhibition, such as temporal cortex, lateral geniculate nucleus (LGN) of the thalamus, and vision cortex [44, 45], while tonic inhibition is mediated via extrasynaptic *α*5- and *δ*-containing GABA_A_Rs [40, 44]. Future research will examine that specific subunits of GABA receptor encode these vision information computations. And it is also worth noting that the role of GABA in the developing CNS is dynamic and variable between brain regions [40, 43, 46]. Another triggering idea is that impaired inhibition comes from activity-dependent synaptic plasticity alteration during developmentally critical periods [43, 47]. Both mouse and *Drosophila* FXS models show impaired critical period plasticity, and early activity is critical for shaping E/I synaptic balance [48–50]. These findings indicate that many mechanisms are to be explored among GABAergic neurons, GABA metabolism, and GABA receptor alteration in FXS.

## 4. Impaired GABAergic Neural Circuits in FXS

Dysfunctional mGluR1/5 signaling in excitatory synaptic circuitry has been considered as one classic mechanism underlying FXS [51–53]. But a main characteristic of the impairment is usually attributed to a failure in the inhibition of the central set or the need for a supervisory system to be involved in the inhibition of predominant manners. The increased excitability of hippocampal and neocortical circuits in FXS, due to dysregulation of glutaminergic neurons, can in turn disrupt the normal actions of inhibitory GABAergic neurons [32, 54]. It has long been known that FXS models also display reduced function in inhibitory GABAergic circuits [55–57]. Specifically, downregulation of GABA_A_ receptor subunits influences both the mRNA and protein levels, which would further increase the excitability of limbic and cortical circuits [39].

FMRP is widely expressed in GABAergic neurons [58, 59], and it is also involved in normal interneuron maturation and function modulation [30, 55, 58]. Recently, it was shown that there were lower expressions of several genes involved in GABA metabolism, including *gad1*, *gat1*, and *gat4*, in the brain of both mouse and *Drosophila* FXS models [15, 58, 60]. It is well known that GABAergic neurons can modulate neurotransmitter release in autocrine or paracrine pattern, via presynaptic GABA_A_ and GABA_B_ receptors [61–63]. It is indicated that dysfunctional GABAergic neurons affect balance of inhibitory/excitatory circuits particularly during early developmental critical periods, via the role of GABA attenuated regulator in FXS models [62, 64].

For now, GABAergic impairments have been reported in FXS models of *Drosophila*, *zebrafish*, and mouse. And GABAergic signaling is essential for regulating neuronal migration, maturation, and circuit formation. Therefore, defects in the GABAergic system are likely to have profound effects on neuron development and circuit work in FXS. Currently, a better understanding of early developmental changes in GABAergic system in FXS would be reckoned as the key insight into the underpinning of the FXS brain. Also, the relationship between GABAergic systems and mGluRs ones, as well as their overlapping plasticity alteration, is taken as the pivotal basement to strengthen a more comprehensive cognition of FXS.

Besides the deficits in learning and memory in these models, one consistent behavioral abnormality they share is altered circadian rhythm behaviors, which potentially mimics the sleep abnormalities seen in patients with fragile X syndrome. Circadian rhythm describes the approximately 24-hour cycles generated by a master pacemaker located in the suprachiasmatic nuclei (SCN) of the anterior hypothalamus of the mammalians and in the ventral lateral neurons (LNvs) of *Drosophila* [65]. Also, the connections between the SCN and other parts of the system are important for the control of circadian rhythms in the central nervous system [66]. Interestingly, it has been shown that the loss of FMRP and FXR2P results in arrhythmicity resulting from inappropriate neuronal communication within the central nervous system [67]. Additionally, the altered expression of the clock component has been observed in FXS animal models [67, 68]. The upregulation of FMRP increases PER1- and PER2-induced BMAL1–NPAS2 transcriptional activity, suggesting that FMRP is required for regulation of circadian behaviors. Thus, *Drosophila* lacking the *fmr1* gene exhibits altered circadian rhythms. Taken together, these results indicate that fragile X-related proteins might be associated with the induction of abnormal sleep patterns in FXS due to alterations in circadian genes; they may also play a critical role in the regulation of circadian output pathways.

Clinical studies have illustrated that melatonin-dependent signaling pathways can impair vigilance, learning, and memory abilities and may be linked to autistic behaviors such as abnormal anxiety responses [69, 70]. Low melatonin levels are related with altered GABAergic system [71]. Furthermore, alterations in the circadian clock mechanism due to abnormal melatonin synthesis can affect the function of GABA neural circuits [70, 72]. Recently, studies using animal models of autism have indicated that clock and clock-related genes may interact in the ASD phenotype and studies using *fmr1* KO mice have implicated clock proteins in sleep alterations in FXS [73]. Under dysfunctional FMRP conditions, GABA activity is altered by disruptions in intracellular signaling. Recent studies have proposed the existence of abnormalities in melatonin secretion and circadian patterns in individuals with FXS with ASD that are likely to be due to excessive signaling via GABA [74]. Furthermore, melatonin is helpful for treating the physical alterations of axons and dendritic spines [75, 76]. In addition, other endocrine hormones, such as oxytocin and insulin, participate GABA neuronal function via abnormal biorhythmic patterns. It is reported that oxytocin-mediated GABA excitatory-inhibitory shift during delivery is abolished in FXS model. During delivery and subsequently hippocampal neurons have elevated intracellular chloride levels and elevated gamma oscillations, which suggests the importance of oxytocin-mediated GABAergic inhibition during the process [77]. Similarly, the insulin-producing cells (IPCs) are crucial for normal insulin release and insulin-signaling in the brain and are sufficient to restore normal circadian behavior in the *Drosophila* FXS model [78]. Moreover, IPCs have been demonstrated to receive inputs from multiple neurotransmitters and hormones, including tachykinin, leptin, GABA, and serotonin [79]. But the specific mechanisms deserve further investigation. In brief summary, alteration of GABA inhibition is not simply linked to amplified mGluR signaling, whereas they both are regulated by circadian clock and circadian genes in depth.

Overall, current issues provide much needed *in vivo* evidence for GABAergic circuit impairments in FXS and set the foundation for future work linking molecular to circuit level to behavioral changes. Addressing altered GABAergic circuit function should lead to more effective treatments for FXS patients.

## 5. Conclusions

In summary, deregulated GABA metabolism, decreased expressions of GABA receptor subunits, and impaired GABAergic neural circuits contribute to abnormal behaviors in FXS. Importantly, it is noteworthy to be studied that circadian clock genes regulate substantial life activities of organism and are related to the process of growth and development in FXS models and patients. Specifically, GABA inhibition is modulated via dysfunctional biorhythmic patterns of endocrine hormones and *fmr1* gene. And better understanding of the GABA neural circuits will support novel therapeutic methods in FXS.
